# Research progress on risk prediction models of physical restraint in the elderly: a narrative review

**DOI:** 10.3389/fragi.2025.1650339

**Published:** 2025-10-28

**Authors:** Shaoyi Tao, Hui Li, Jian Huang, Juan Li

**Affiliations:** ^1^ Department of Nursing, The Second People’s Hospital of Hefei, Hefei Hospital Affiliated to Anhui Medical University, Hefei, China; ^2^ Department of Nursing, People’s Hospital of Zongyang County, Tongling, China; ^3^ Department of Thoracic Surgery, The First Affiliated Hospital of USTC, Division of Life Sciences and Medicine, University of Science and Technology of China, Hefei, China; ^4^ Department of Nursing, The First Affiliated Hospital of USTC, Division of Life Sciences and Medicine, University of Science and Technology of China, Hefei, China

**Keywords:** elderly, physical restraint, risk prediction models, risk assessment, geriatric care

## Abstract

This review presents the advancements in research on risk prediction models for physical restraint among the elderly. As the global population ages, the issue of physical restraint in older adults has become increasingly prominent, making accurate risk prediction essential for enhancing their quality of life. Current Status: Physical restraint rates exhibit marked regional disparities (e.g., 84.9% in Spain vs. 1.9% in the US). Key risk factors include age ≥75, dementia, and agitation. Machine learning models achieve higher accuracy than traditional statistical approaches, but hybrid models better balance precision and interpretability. Future Directions: (1) Developing real‐time monitoring systems via sensor technology; (2) Establishing ethical frameworks for model deployment through clinician-data scientist partnerships; (3) Implementing validated tools in clinical settings to minimize restraint use. Finally, the review emphasizing the need for improved methodologies and the integration of interdisciplinary approaches to better address this complex issue.

## 1 Introduction

The rising use of physical restraints in elderly healthcare, while aimed at safety, poses ethical concerns and risks, such as falls and psychological trauma; thus, accurate risk prediction models are crucial for identifying at-risk patients and personalizing interventions, supported by advancements in data analytics and machine learning (Kooken et al., 2023). Research shows that restraints are common yet often applied without understanding their risks, highlighting the need for healthcare professionals to prioritize patient autonomy (Ocagli et al., 2021). Developing robust prediction models with machine learning to analyze patient characteristics can help tailor interventions, reduce restraint use, and improve outcomes. Additionally, integrating personalized care, such as therapeutic activity kits for dementia patients, can minimize restraint needs and align with ethical practices (Higgs et al., 2020). In an evolving healthcare landscape, emphasizing evidence-based, patient-centered care through effective prediction models for elderly restraint use is essential for fostering a respectful environment. This review synthesizes existing research, identifies knowledge gaps, and proposes directions for future studies.

## 2 Methods

This article adopts a narrative review approach to sort out the existing literature on risk prediction models for physical restraint in the elderly. Based on the biopsychosocial model (BPSM), this review integrates biological, psychological, and sociological factors to analyze restraint risks. A literature search was conducted through major databases such as PubMed and Web of Science. The keywords used included “physical restraint”, “risk prediction”, “elderly” and “machine learning”. The inclusion criteria focused on studies exploring prediction models; these comprised original research, systematic reviews, and meta-analyses, while news or commentary articles were excluded. Data extraction included model types, performance metrics, key predictive factors (such as age and cognitive function), intervention strategies, and the impact of environmental factors.

## 3 Definition and classification of physical restraints for the elderly

### 3.1 Definition and common forms of physical restraint

Physical restraints in healthcare limit patient movement to prevent injury. Common types include bed rails, restraint belts, and fixed seating devices. Bed rails prevent falls but can cause risks like entrapment, especially for cognitively impaired elderly patients (Hakverdioğlu et al., 2023; Everitt and Bridel-Nixon, 1997). Restraint belts secure patients but may lead to discomfort or helplessness, increasing agitation in vulnerable groups. Fixed seating devices provide support but can restrict mobility and independence, essential for older adults’ wellbeing (Edlich et al., 2003).

**FIGURE 1:**
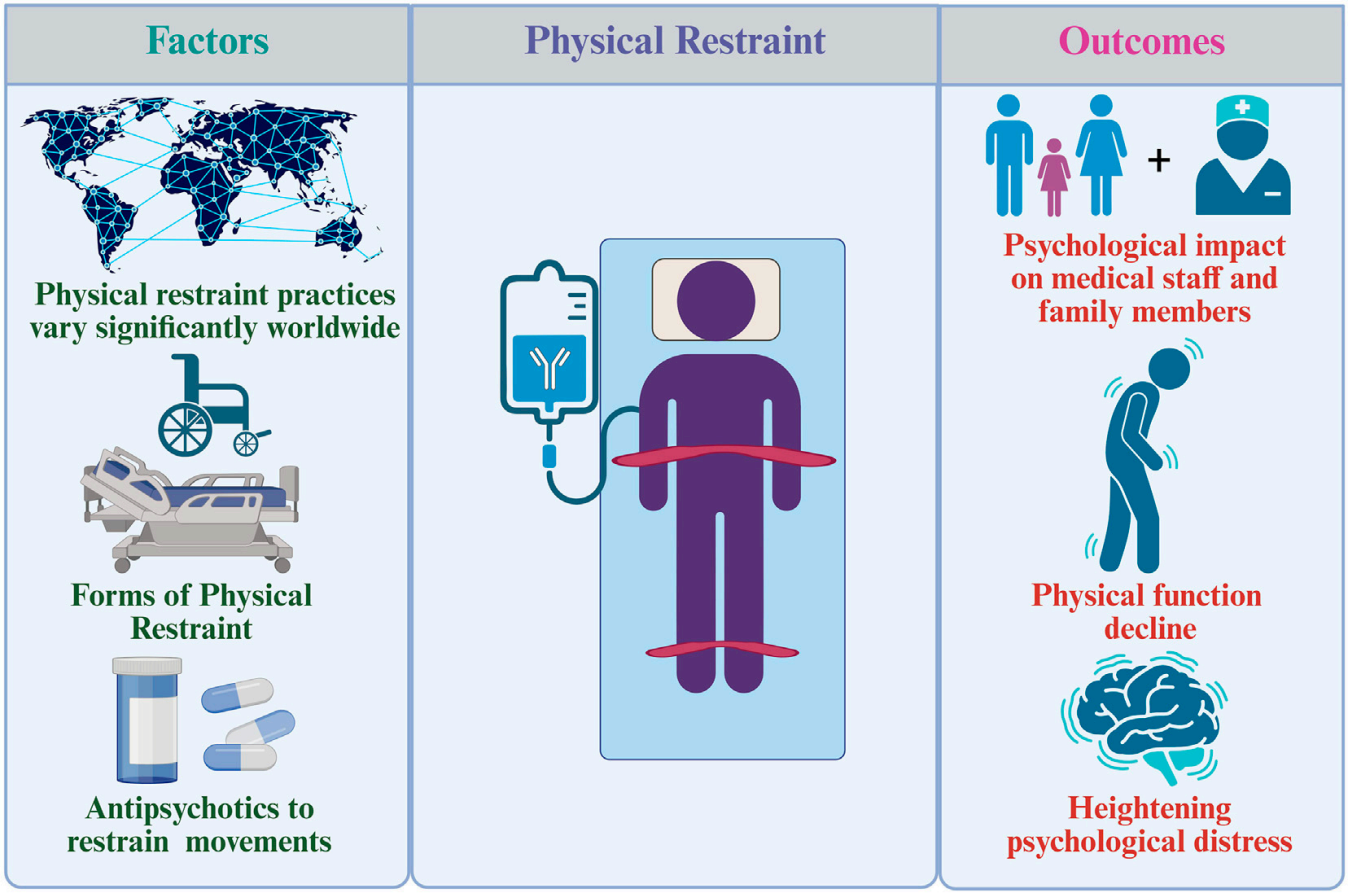
Comprehensive overview of physical restraint.

The potential hazards associated with different types of physical restraints vary significantly. For instance, the use of bed rails can lead to a false sense of security, resulting in inadequate monitoring of patients who may still be at risk of falling (Hamers and Huizing, 2005). Studies have shown that while bed rails can reduce fall rates, they may inadvertently increase the likelihood of falls due to patients attempting to climb over them or becoming entangled (Wang et al., 2025). Similarly, restraint belts, while intended to enhance safety, can lead to severe complications such as pressure ulcers or impaired circulation if not monitored correctly (Grimes et al., 2022). The psychological impact of restraints should also be considered; patients may experience increased anxiety or agitation when restrained, particularly if they perceive the restraints as a loss of autonomy (Zhu et al., 2022). Fixed seating devices can support patients with mobility restrictions but may lead to a sedentary lifestyle and health issues like muscle atrophy; careful assessment of individual needs is essential for balancing benefits and harms while prioritizing patient dignity and autonomy (Choudhary et al., 2022). Figure 1 illustrates a comprehensive overview of the physical restraints.

### 3.2 The use of drugs to restrain elders’ movements

Chemical restraint involves using pharmacological agents to limit a patient’s movement, especially in managing agitation or behavioral issues in elderly patients, with common medications including antipsychotics like haloperidol and atypical ones such as olanzapine, which modulate neurotransmitter systems to induce calmness (Mesraoua et al., 2023). Ethical considerations regarding chemical restraints are crucial, emphasizing the principle of least restraint, which promotes non-pharmacological interventions like therapy and staff training before using such measures. The decision to administer chemical restraints requires thorough patient assessment, weighing benefits and risks, and obtaining informed consent. While they can be necessary for managing acute behavioral issues in elderly patients, their use must prioritize patient safety and dignity (Rosano et al., 2021).

### 3.3 Environmental constraints

Environmental constraints can limit elderly individuals’ mobility and wellbeing, manifesting as restricted areas, inadequate services, or unsafe conditions, which may lead to isolation, depression, and anxiety, impacting their overall health (Khoo et al., 2022; Li et al., 2024). Moreover, the presence of environmental constraints can lead to a reliance on physical restraints as a preventive measure in care settings, raising ethical concerns regarding autonomy and dignity. The use of physical restraints may be viewed as a necessary intervention to ensure safety; however, it can also lead to negative physical and psychological outcomes for the elderly, such as increased agitation, depression, and a decline in physical function (Hakverdioğlu et al., 2023; Okuno et al., 2021). Therefore, it is essential to explore alternative strategies that focus on modifying the environment to reduce the need for restraints while promoting independence and quality of life among older adults. This approach necessitates a shift towards creating supportive environments that accommodate the unique needs of the elderly, thereby minimizing the risks associated with both physical restraints and environmental constraints ((Li Y. et al., 2022; Välimäki et al., 2022). Understanding environmental constraints and their interplay with other limitations is crucial for creating effective risk prediction models for the elderly, enhancing care quality, and supporting their autonomy. Future research should explore these constraints further to develop innovative solutions that ensure safety and dignity (4, (Li et al., 2024).

## 4 Epidemiological characteristics of physical restraint in the elderly

### 4.1 Global prevalence data comparison

The use of physical restraints among the elderly varies significantly globally, influenced by cultural, ethical, and systemic factors; for example, Spain has a high prevalence of physical restraints (84.9%) in nursing homes, while the U.S. has only 1.9% (Hakverdioğlu et al., 2023). In Asian countries like China, family caregivers, guided by Confucian values, often prioritize restraint for safety, leading to higher usage in home care. Restraint types differ regionally, with bed rails being prevalent in Singapore (98%) *versus* Germany (4.7%) (Hakverdioğlu et al., 2023). Cultural perceptions influence practices, as Western countries push for minimal restraint use, while Asian cultures may emphasize familial protection (Ma et al., 2023). Systemic issues like staffing and training also affect prevalence, suggesting a need for culturally sensitive guidelines and training to respect elderly autonomy and safety.

### 4.2 Differences in restraint use by institution type

The use of physical restraints in elderly care varies significantly across different institutional settings, including hospitals, nursing homes, and home care environments. In hospitals, restraints are often employed as a last resort to prevent patients from harming themselves or others, particularly in acute care settings where patients may exhibit disruptive behavior due to delirium or severe cognitive impairment (Liang and Huang, 2023). Research indicates that the prevalence of restraint use in hospitals can be influenced by factors such as staff training, hospital policies, and the availability of alternative interventions (Medina Ortega et al., 2024; Thunborg et al., 2021). Conversely, nursing homes tend to have higher rates of restraint use, often justified by concerns over falls and the need for safety among residents with cognitive impairments, such as dementia (Li Y. et al., 2022; Pu et al., 2023). In these settings, the decision to use restraints is frequently influenced by the staff’s knowledge, attitudes, and practices regarding restraint, which can vary widely based on institutional policies and culture (Carr et al., 2021). Home care involves family caregivers making decisions about restraint use influenced by cultural factors and environment, with Chinese caregivers facing moral dilemmas in dementia care due to traditional values, lack of guidelines, and varying perceptions of safety regarding restraints (Ma et al., 2023).

Institutional policies are crucial in shaping the rates of restraint use across these different environments. In hospitals, guidelines often emphasize the need for a thorough assessment before implementing restraints, alongside a focus on alternative strategies to manage agitation or disruptive behavior (Sharifi et al., 2021). In contrast, nursing homes may lack such rigorous policies, leading to a more routine application of restraints as a preventive measure against falls or injuries (Li Y. et al., 2022). The cultural context also plays a significant role, as societal attitudes toward aging and caregiving can influence how restraints are perceived and implemented across different settings (Ma et al., 2023).

Overall, the differences in restraint use across various institutions underscore the need for standardized guidelines that consider the unique challenges and dynamics of each environment. Enhanced training for staff, improved communication among caregivers, and the development of institutional policies that prioritize person-centered care can help reduce the reliance on physical restraints and promote safer, more dignified care for elderly individuals (Medina Ortega et al., 2024; Pu et al., 2023).

## 5 Risks and adverse consequences of physical restraint

The use of physical restraints in the elderly poses serious health risks, including muscle atrophy, pressure ulcers, and falls, which significantly affect mobility and physical function; prolonged restraint worsens these issues and increases the likelihood of complications such as infections and hospitalization, while also heightening psychological distress, thus diminishing the quality of life (Meade et al., 2020; Li B. et al., 2022). Physical restraints have a direct negative impact on functional independence, as they significantly limit mobility and, in turn, reduce an individual’s ability to perform self-care tasks such as feeding, toileting, and dressing. When physical function deteriorates due to the use of restraints, individuals become increasingly dependent on others for basic activities of daily living (ADL). Research indicates that irreversible declines in the ability to perform ADL can occur after just 72 h of continuous restraint use, highlighting the urgent need to reconsider the use of restraints in care settings to preserve the autonomy and functional capabilities of individuals (Soeno et al., 2021; Foebel et al., 2016). The physical restraints imposed on patients can also have significant psychological effects on healthcare professionals and family members alike. The first issue is the moral dilemma, where individuals are fully aware that the constraints imposed may be harmful, yet they feel compelled to enforce them, which can ultimately lead to feelings of professional burnout; the second issue arises from decision-making pressure, where the demands of family members for constraints conflict with the autonomy of the patient, resulting in heightened anxiety; finally, the third issue stems from a sense of helplessness caused by a lack of adequate training, leaving individuals feeling unprepared and overwhelmed in their roles (Kong et al., 2017; Kavumpurath et al., 2023).

## 6 The theoretical basis of risk prediction models

### 6.1 Application of the biopsychosocial model

The Biopsychosocial Model (BPSM) integrates biological, psychological, and social factors to understand health outcomes in elderly care, guiding the selection of predictive factors for risks associated with physical restraints. BPSM serves as the cornerstone for understanding the issue of physical restraint in the elderly. By analyzing the nonlinear interactions among biological, psychological, and social factors, the BPSM offers a systematic approach to reducing the use of physical restraints (McKay et al., 2012; Wright et al., 2007). This holistic approach helps healthcare professionals identify predictors like cognitive impairment, emotional distress, and social support quality that influence an elderly individual’s health, emphasizing the importance of how these dimensions interact, such as in cases of dementia where cognitive decline, anxiety, and lack of social support increase restraint risk (Tang et al., 2021; Cui et al., 2023). The interactions among biological, psychological, and social factors are essential for understanding the use of physical restraints, as conditions like frailty and anxiety can lead to challenging behaviors, while supportive networks can offer alternatives, highlighting the need for comprehensive interventions like enhancing social support and psychological therapies to reduce restraints in the elderly (Cui et al., 2023; Tang and Wang, 2023). The BPSM leads to effective, tailored interventions for elderly patients by addressing their unique biological, psychological, and social needs, reducing reliance on physical restraints and improving healthcare staff’s decision-making through ongoing education (Tang et al., 2021; Tang and Wang, 2023).

### 6.2 The role of vulnerability theory in predictive models

The relationship between the accumulation of frailty and the risk of restraint in elderly individuals has garnered increasing attention in geriatric research. Vulnerability theory posits that frailty is not merely a consequence of aging but a complex interplay of biological, psychological, and social factors that accumulate over time, leading to increased susceptibility to adverse health outcomes, including restraint (Stratidaki et al., 2024). Studies have shown that elderly individuals exhibiting higher levels of frailty are at a greater risk for being physically restrained in clinical settings, as caregivers may perceive them as less capable of self-care or more prone to falls and injuries (Huang et al., 2022; Dong et al., 2025). This accumulation of frailty can manifest through various indicators, such as decreased physical strength, cognitive impairment, and social isolation, all of which contribute to the likelihood of restraint being deemed necessary for safety reasons.

Quantifying frailty indicators for risk prediction is essential for developing effective interventions and care strategies. Researchers have employed various methodologies to operationalize frailty, often utilizing frailty indices that aggregate multiple health deficits to provide a comprehensive assessment of an individual’s vulnerability (Yu and Yu, 2025). These indices typically include factors such as mobility limitations, nutritional status, and the presence of chronic diseases, which together create a profile of frailty that can be used to predict the risk of restraint. For instance, the Fried Frailty Phenotype is a widely recognized model that assesses frailty based on five criteria: unintentional weight loss, exhaustion, low physical activity, slow walking speed, and weakness (Huang et al., 2022). Moreover, machine learning algorithms have shown promise in enhancing the predictive accuracy of frailty risk assessments. Recent studies have applied advanced statistical techniques to analyze large datasets, identifying complex patterns and interactions among frailty indicators that traditional models may overlook (Cao et al., 2025; Liu et al., 2024).

## 7 Overview of existing risk prediction models

### 7.1 Traditional statistical models

Traditional statistical models, such as logistic regression and Cox proportional hazards models, have been widely used in the medical field for risk prediction, particularly concerning elderly populations. Logistic regression is particularly valuable for binary outcomes, allowing researchers to understand the relationship between one or more predictor variables and the likelihood of a certain event occurring, such as falls or hospitalizations in older adults (Cai et al., 2009). However, these traditional models have limitations, including reliance on linear relationships and proportional hazards, often failing to capture complex interactions in predicting MACE in elderly patients (Gupta et al., 2025). Despite these limitations, traditional statistical models remain foundational in risk prediction, providing a baseline for comparison with newer methodologies. Their interpretability and established frameworks make them valuable tools in clinical practice, especially when combined with other predictive techniques to enhance overall accuracy and reliability in risk stratification for elderly populations.

### 7.2 Machine learning models

The application of machine learning (ML) models in predicting the risk of physical restraints in elderly patients has gained traction due to their ability to analyze complex datasets and identify patterns that may not be evident through traditional statistical methods. Among the various machine learning algorithms, Random Forest (RF) and Support Vector Machines (SVM) have emerged as prominent tools in this domain (Ogutu et al., 2011; Siemers and Bajorath, 2023). RF, a tree-based ensemble learning method, excels at handling high-dimensional data and is particularly effective in managing the variability and nonlinearity commonly found in clinical datasets. In studies predicting outcomes such as postoperative delirium and fall risk in elderly patients, RF has demonstrated exceptional performance. For instance, in hypertension prediction research, RF achieved an accuracy of 0.90 (Turnbull et al., 2024), while in athletic identification studies, it reached an accuracy of 0.92 (Estrella and Capdevila, 2025), demonstrating high accuracy and robust predictive capabilities. Furthermore, in sex estimation tasks, RF achieved an accuracy of 73% (Syed et al., 2025), further confirming its advantages in clinical prediction. On the other hand, SVM, which excels at finding optimal hyperplanes to separate different classes in high-dimensional spaces, has also shown promising results in various applications, including predicting suicide risk and other adverse outcomes in elderly populations. For example, in frailty identification studies, SVM achieved an AUROC of 78.05% (Chou et al., 2024); in athletic identification research, it reached an accuracy of 0.84 (Estrella and Capdevila, 2025); and in music familiarity classification, it achieved an accuracy of 67% (Kim et al., 2024), highlighting its applicability across different scenarios. Comparative analysis of these algorithms indicates that while both RF and SVM can achieve high predictive accuracy, their performance may vary depending on the specific characteristics of the dataset and the nature of the prediction task. For example, in hypertension prediction studies, RF’s accuracy (0.90) was significantly higher than SVM’s (0.57) (Turnbull et al., 2024), and in sex estimation research, RF’s accuracy (73%) also outperformed SVM’s (67%) (Syed et al., 2025), suggesting that RF often surpasses SVM in terms of AUC, indicating better overall model performance. However, SVM demonstrates effectiveness in scenarios with smaller sample sizes or when data is non-linearly separable. For instance, in antioxidant activity prediction, both SVM and RF achieved accuracy exceeding 0.9 (Jung et al., 2024), showcasing SVM’s versatility in handling diverse clinical datasets. Future research should focus on refining these models, incorporating larger and more diverse datasets, and ensuring their clinical applicability through rigorous external validation. Table 1 summarizes the performance of various machine learning models across multiple application domains.

**TABLE 1:** Performance comparison of machine learning models.

Application domain	Machine learning model	Performance metric	Value	Source
Hypertension Prediction	Random Forest (RF)	Accuracy	0.9	47
	Support Vector Machine (SVM)	Accuracy	0.57	
Sex Estimation	Random Forest (RF)	Accuracy	73%	49
	Support Vector Machine (SVM)	Accuracy	67%	
	Linear Discriminant Analysis (LDA)	Accuracy	65%	
Frailty Identification	Support Vector Machine (SVM)	AUROC	78.05%	50
Antioxidant Activity Prediction	Random Forest (RF)	Accuracy	>0.9	52
	Support Vector Machine (SVM)	Accuracy	>0.9	

Area under the receiver operating characteristic curve (AUROC).

### 7.3 Mixed models and ensemble methods

The integration of clinical rules with data-driven methods using mixed models and ensemble techniques has significantly advanced risk prediction in medical conditions, particularly for the elderly. Mixed models, especially linear mixed models (LMMs), effectively manage hierarchical data and individual variability, enhancing prediction accuracy by adjusting for confounding variables (Tirrell et al., 2018). Recent studies highlight the superiority of mixed models in predicting chronic disease outcomes and the benefits of ensemble methods like random forests and gradient boosting in improving prediction stability and generalizability for elderly care, ultimately supporting personalized interventions and optimizing healthcare for aging populations (Ng et al., 2018; Griesbach et al., 2021). Table 2 summarizing the various risk prediction models.

**TABLE 2:** Strengths and limitations of each model.

Model type	Key examples	Theoretical strengths	Theoretical limitations
Traditional Statistical Models	• Logistic Regression • Cox Proportional Hazards	• Proven interpretability of predictor-outcome relationships • Established frameworks for binary outcomes (e.g., restraint use) • Clinically accessible implementation	• Assumes linearity, failing to capture complex interactions • Requires proportional hazards assumption (Cox models) • Prone to overfitting and multicollinearity with moderate predictive accuracy
Machine Learning (ML) Algorithms	• Random Forest (RF) • Support Vector Machines (SVM)	• Handles high-dimensional data efficiently • Detects non-linear patterns and interactions missed by traditional models • Superior predictive accuracy (e.g., RF for feature importance) • SHAP methods enhance interpretability	• “Black box” nature complicates clinical trust • Requires large datasets for training and validation • Risk of overfitting without rigorous cross-validation
Hybrid Models	• Linear Mixed Models (LMMs) • Ensemble Approaches (e.g RF + Clinical Rules)	• Combines clinical expertise with data-driven insights • Adjusts for individual variability/hierarchical data (LMMs) • Improves stability and generalizability (ensemble methods) • Supports personalized intervention planning	• Complex integration of clinical/data sources increases deployment difficulty • Validation protocols remain inadequately standardized • Limited real-world testing in diverse elderly cohorts

## 8 Key predictive factor analysis

### 8.1 Demographic and clinical characteristics

Key predictive factors for restraint use include age, gender, and comorbidities; older adults face higher risks of functional impairments and cognitive decline, leading to increased restraint, with those 75 and older particularly affected (Ji et al., 2025). Women are also at greater risk due to health conditions, while comorbidities like dementia elevate the likelihood of restraint among the elderly with multiple chronic issues (Lorenzo et al., 2014; Sajatovic et al., 2002). The predictive efficacy of factors like comorbidities varies by population; urban areas show higher restraint use linked to conditions like hypertension and diabetes, while rural settings reflect different patterns influenced by healthcare access and caregiver methods. Additionally, the interplay of demographic and clinical traits can complicate predictions, as elderly individuals with both cognitive impairment and physical disabilities face greater restraint risks, highlighting the need for nuanced risk assessments (Boateng et al., 2023; Leinaweaver, 2022; Hilberger et al., 2024).

### 8.2 Cognitive and behavioral factors

Cognitive assessment tools are vital for predicting restraint risks in the elderly, as lower cognitive scores relate to behaviors like wandering and agitation, which lead to restraint use. Behavioral symptoms significantly predict restraint application, creating dependency and highlighting the need for cognitive assessments to identify at-risk individuals, allowing caregivers to maintain patient dignity. Elderly individuals with cognitive impairments often show agitation and wandering, prompting restraint use, thus emphasizing the importance of behavioral assessments alongside cognitive evaluations (Vintimilla et al., 2020). These insights stress the need for understanding cognitive function and behavior in predicting restraint use, enabling healthcare providers to identify at-risk individuals and implement interventions that respect elderly patients’ autonomy and dignity while reducing restraint use (Hakverdioğlu et al., 2023; Vintimilla et al., 2020; Lam et al., 2017; Wood, 2025).

## 9 Future research directions

The implementation of predictive models for physical restraints in elderly care is essential for evaluating effectiveness and translating research into practice, requiring a systematic framework that assesses model accuracy and applicability through validation studies. Future studies should focus on establishing a multidisciplinary framework to address the integration of ethical considerations within predictive models. This involves forming collaborative teams that include data scientists, clinical practitioners, and ethicists to jointly determine the weighting of ethical factors. Additionally, ethical principles such as autonomy protection and harm minimization need to be translated into quantifiable clinical indicators. A dynamic calibration mechanism should also be developed to continuously refine these weightings based on real-time feedback from clinical practice. Such efforts will ensure that predictive models maintain scientific rigor while systematically incorporating ethical dimensions, thereby enhancing both their applicability and alignment with clinical ethics. The direction of technical integration research needs to address the issue of insufficient dynamic prediction capabilities of existing models. Current research mostly relies on static data (such as electronic health records), lacking real-time monitoring of dynamic indicators, such as the risk of falls and agitation. By combining wearable devices with AI algorithms, it is possible to overcome the temporal limitations of traditional statistical models, such as logistic regression. Interdisciplinary collaboration aims to overcome barriers to the clinical translation of existing predictive models. One major issue is that only 12% of these models have been adopted in clinical settings. This low adoption rate is primarily due to the lack of association between model outputs and intervention strategies, such as environmental modifications. We propose a prediction-intervention linkage framework that integrates nursing ethics, rehabilitation medicine, and fills the gap in translating predictive models into clinical practice. The optimization of the ethical framework addresses the constraint paradox—a situation often overlooked in the literature—where imposing constraints leads to a decline in specific behaviors, such as reduced compliance or engagement. Based on the BPSM theory, we emphasize the need to incorporate “autonomy protection,” including measures like prioritizing alternative interventions, into the predictive indicators. This approach extends beyond the current research paradigm, which focuses solely on risk probabilities. Evaluating the long-term impact of these models on clinical outcomes is crucial for demonstrating their value and securing resources for sustained initiatives, ultimately improving elderly care.

## 10 Conclusion

Recent advancements in risk prediction models for elderly physical restraints highlight ethical implications and clinical challenges. While progress is evident, issues with predictive validity persist, necessitating improvements through advanced techniques. Collaboration between researchers and clinicians is essential for practical applicability, and ethical frameworks must guide restraint decisions. Future research should leverage technology for real-time data and focus on successful implementation to enhance elderly care quality. Establishing a reliable risk prediction system for older adults aims to minimize physical restraints and improve their quality of life through ethical practices and innovation in healthcare.
